# Projected Impact of Climate Change on Hydrological Regimes in the Philippines

**DOI:** 10.1371/journal.pone.0163941

**Published:** 2016-10-17

**Authors:** Pamela Louise M. Tolentino, Ate Poortinga, Hideki Kanamaru, Saskia Keesstra, Jerry Maroulis, Carlos Primo C. David, Coen J. Ritsema

**Affiliations:** 1 National Institute of Geological Sciences, University of the Philippines, Diliman, Quezon City, Philippines 1101; 2 Soil Physics and Land Management (SLM), Wageningen University and Research Center, P.O. Box 47, 6700 AA Wageningen, The Netherlands; 3 Water Insight, Postbus 435, 6700 AK Wageningen, The Netherlands; 4 Climate, Energy and Tenure Division, FAO, Largo Terme di Caracalla 00153 Rome, Italy; 5 International Centre for Applied Climate Science (ICACS), University of Southern Queensland (USQ), Toowoomba QLD 4350, Australia; University of Vigo, SPAIN

## Abstract

The Philippines is one of the most vulnerable countries in the world to the potential impacts of climate change. To fully understand these potential impacts, especially on future hydrological regimes and water resources (2010-2050), 24 river basins located in the major agricultural provinces throughout the Philippines were assessed. Calibrated using existing historical interpolated climate data, the STREAM model was used to assess future river flows derived from three global climate models (BCM2, CNCM3 and MPEH5) under two plausible scenarios (A1B and A2) and then compared with baseline scenarios (20th century). Results predict a general increase in water availability for most parts of the country. For the A1B scenario, CNCM3 and MPEH5 models predict an overall increase in river flows and river flow variability for most basins, with higher flow magnitudes and flow variability, while an increase in peak flow return periods is predicted for the middle and southern parts of the country during the wet season. However, in the north, the prognosis is for an increase in peak flow return periods for both wet and dry seasons. These findings suggest a general increase in water availability for agriculture, however, there is also the increased threat of flooding and enhanced soil erosion throughout the country.

## Introduction

Due to its geographical setting, the Philippines is naturally vulnerable to hydrometeorological hazards such as tropical cyclones, flooding, droughts, and rain-induced landslides. These environmental hazards are aggravated by human activities such as deforestation and improper land use planning. Moreover, the Philippines is one of the most vulnerable countries to the impacts of climatic change [1] due to its high level of risk exposure and limited resources for adaptation [2].

Furthermore, climate change represents a serious threat to the Philippines which is heavily reliant on agriculture for food security and economic growth. The livelihood of millions is threatened by the potential effects of climate change as current agricultural practices are adapted primarily to the prevailing climate. Under predicted climate change, current agricultural practices may become unsustainable due to changing rainfall patterns or temperature rises that reduce the viability of certain crop types. Therefore, an understanding of future seasonal variability in rainfall patterns and thus hydrological regimes under the impacts of climate change, is critically important in deciphering the ability of catchments to reliably supply irrigation water.

In order to forecast the impact of climate change, the Intergovernmental Panel of Climate Change (IPCC) has produced various future scenarios from which greenhouse gas emissions (GHG) are estimated and global climate models (GCMs) developed. Some studies have used the data to assess the effects of a warmer climate on flood risk at a global or continental scale [3, 4]. Despite the plethora of GCMs running numerous scenarios, these GCMs are however too coarse to assess the impact of climate change at a country scale let alone for examining specific hydrological basins within a country.

Consequently, the Food and Agriculture Organization (FAO) developed an integrated suite of climate models for assessing the impact of climate change on agriculture at the national level. The MOdelling System for Agricultural Impacts of Climate Change (MOSAICC) is a generic methodology designed to assess the impact of climate change on agriculture, by incorporating crop yields, water resources, macro-economic and downscaled climate data.

The MOSAICC framework was deployed in this study to assess the potential impact of climate change on river flows under various climate scenarios up to 2050 for the Philippines. A time-series of historical interpolated downscaled climate data of hydrological simulations for model calibration was also used. Data from three GCMs (BCM2, CNCM3, MPEH5) with two likely scenarios each (A2 and A1B), were then input into the hydrological model.

## Materials and Methods

### Study area

**Fig 1:**
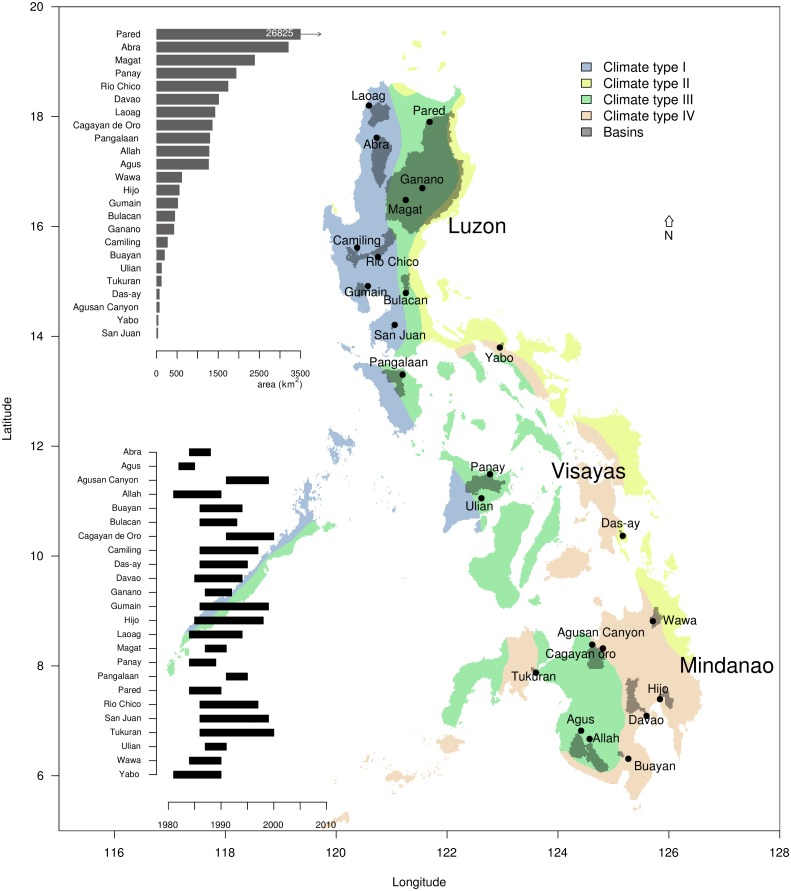
A map of the Philippines showing the coloured climatic type (I-IV) according to the Coronas classification [5–7]. The darker shaded areas on the map highlight the locations of the 24 basins that were included in this study, while the named points represent the basin outlets. The bar plot in the upper left corner represents the ranked total area of each of the 24 basins, while the graph in the lower left corner shows the period of available measured hydrological data used for model calibration for each basin.

The Philippines (Fig 1) is an archipelago made up of >7,000 islands with a total area of 300,000 km^2^. There are three main island groupings: Luzon in the north (141,000 km^2^), Visayas in the middle (57,000 km^2^) and Mindanao in the south (102,000 km^2^). The Philippines contains 421 principal river basins, 18 of which are considered major river basins, each with a minimum watershed area of 1,400 km^2^. These river basins are important sources of freshwater resources for meeting agricultural, commercial and domestic demands.

The climate of the Philippines can be divided into four distinct categories using the modified Coronas classification [5–7], as shown in Fig 1. Climate Type I is characterized by a distinct dry period from November to April and a wet season from May to October. Type II results in rainfall being evenly distributed throughout year, but with a very pronounced rainy season from November to January. In Type III, the seasons are not very pronounced, but a relatively dry period prevails from November to April. Finally, in Type IV, precipitation is evenly distributed throughout the year. In this analysis, we used the modified Coronas classification as the basis of making the distinction between dry and wet months.

To assess the impact of climate change on the hydrological regime at the country scale, various river basins were selected. This was done on the basis of catchment size, climate type, the availability of historical streamflow data and their importance to agricultural production. A total of 24 basins were selected, which represent sub-basins of the 18 major river basins in the Philippines and which are located within in major agricultural provinces. These basins and their outlets are shown in Fig 1. The basins selected include: 12 in Luzon, 3 in Visayas and 9 in Mindanao, with catchment sizes ranging from the San Juan River at 37.82 km^2^ to Pared River at 26,825 km^2^ (refer to barplot in Fig 1).

### Climate models

To obtain reliable estimates of the likely impacts of climate change on the hydrological regime in the Philippines, determination of the most appropriate GCMs was needed. A further consideration is that GCMs vary both in their sensitivity to different levels of the atmosphere (from surface to 200hPA) and in their parameterization schemes. Downscaling was performed using the results from the sensitivity analysis provided by Manzanas (2015).

The MOSAICC framework, was designed to host a variety of GCMs. In this study, three GCMs appear to be the most effective at simulating climate for the Philippines: ECHAM5/MPI-OM (MPEH5) developed at the Max Planck Institute for Meteorology; BCCR-BCM2.0 (BCM2) from the Bjerknes Centre for Climate Research, and CNRM-CM3 (CNCM3) developed by the Météo-France (Centre National de Recherches Météorologiques). Furthermore, these GCMs are well documented by the Program for Climate Model Diagnosis and Intercomparison (PCMDI) [8] and IPCC [9].

Based on the four Special Reports on Emissions Scenarios (SRES) from the IPCC Fourth Assessment Report (AR4), two likely scenarios were selected to simulate future climate change in the Philippines. The first SRES scenario (A1B) assumes very rapid economic growth, a global population that peaks in mid-century and the rapid introduction of new and more efficient technologies. The second scenario (A2) represents the negative extremes of high population growth, slow economic development and slow technological change. The two scenarios for the period 2010-2050 were compared with simulations of the 20th century (20C3M; referred to as baseline hereafter) which included the period 1971-2000. Calibration of the hydrological model was done using ERA-Interim (1979-2010), the latest global atmospheric reanalysis by the European Centre for Medium-Range Weather Forecasts [10].

Climate data used in this study include precipitation (P) and potential evapotranspiration (PET), where the latter was derived from maximum (Tmax) and minimum (Tmin) temperatures [11]. Basconcillo et al. (2015) [12] statistically downscaled three GCMs under the Coupled Model Intercomparison Project Phase 3 (CMIP3) via the FAO-MOSAICC Portal (http://mosaicc.da.gov.ph). ERA-Interim (1979-2010) is the reanalysis dataset used to generate climate data in the absence of actual climate observations. The quasi-observations were used as predictors for calibrating statistical downscaling models, which Manzanas et al., (2015) [13] attested to its better performance compared to the JRA-25 (1.125° x 1.125°) when compared with actual observations in the Philippines. ERA-Interim data was also used as quasi-observational inputs into the hydrological model because of its spatial and temporal homogeneity compared to the Philippines weather station observations that contained many missing values. The atmospheric elements used as predictors are meridional/zonal wind (U/V), specific humidity (Q) and temperature (T). The identified set of predictors used for downscaling are—U850, Q850, T1000—for Tmin and Tmax and—U850, U300, Q850, and T1000—for precipitation. The study downscaled these three variables at the weather station level using the three selected GCMs. There are 47/33/36 Philippine Atmospheric Geophysical and Astronomical Services Administration (PAGASA) stations for precipitation/Tmin/Tmax, respectively. The study also spatially interpolated the station-level downscaled climate data using the Analyse Utilisant le RELief pour l'HYdromt́éorologie (AURELHY) technique to obtain 10 km-gridded data for the whole country.

### Hydrological model

The hydrological model STREAM (Spatial Tools for River basins and Environment and Analysis of Management options) was used in this study. STREAM is a spatially distributed GIS-based rainfall-runoff model, specifically suited to assess river flows in data scarce environments, as it relies on a water balance for stream flow estimations. The model, developed by Aerts et al., (1999) [14] is optimized for the analysis of the hydrological impact of land use and climate change in river basins. The model has proven to give reliable results in numerous other studies in various locations and climatic regimes [15–20].

STREAM solves the water balance using a gridded landscape in order to estimate stream flows. For each basin, derived from a digital elevation map (DEM), the accumulated runoff and groundwater storage was calculated on a monthly basis. To maintain important topological features in the digital terrain model while keeping calculation times acceptable, the downscaled climate data was resized to the same spatial resolution of the DEM at approximately 1 km. No additional interpolation was applied in this step.

In situ streamflow measurement data were used for model calibration. Data were digitized from physical records that contained daily runoff, while temporal coverage of the data series varied for each of the basins. Consecutive years with no missing data were selected for model calibration, to highlight the difference in temporal coverage. For instance, Fig 1 shows a temporal resolution of 3 years for the Agus basin, while for the Tukuran basin it is 14 years. As discharges were manually measured, quantifying the accuracy of the data with sufficient precision was an issue. As such, the degree of accuracy of records were categorized as “excellent”, “good”, “fair”, or “poor” using the following convention: “Excellent” means about 95% of daily discharges are within ±5% difference of the actual gauge height vs height computed from the rating curve; “Good” is within ±10%; and “Fair” is within ±15%; while “Poor” means daily discharges are below the 15% “Fair” accuracy. The median, mean and distributions of monthly discharge estimates were used for model calibration to account for missing data and variability in data quality.

Hydrological model calibration was done to ensure measured monthly distributions are in agreement with the simulated ones. *In situ* streamflow measurements were used as a reference to validate the performance of the model. These calibrated model results were then used as a reference. Two parameters were manually adjusted to define the groundwater fraction and flow velocity using the precipitation and PET data from the ERA-interim dataset. Due to limited measured river flow data and the relatively coarse resolution of input data, modelled discharge distributions were compared with monthly distributions of measured data, rather than comparing exact periods. The model performance was evaluated by determining the coefficient of determination in comparing the monthly medians of the measured and modelled data and the volumetric efficiency [21]. The volumetric efficiency was calculated using Eq 1, where *VE* denotes the volumetric efficiency, and *Q*_*m*_ and *Q*_*o*_ the modelled and observed discharge, respectively, where 1 indicates a perfect score.

$$VE=1-\frac{\sum |Q_{m}-Q_{o}|}{\sum Q_{o}}$$ (1)

### Data analysis method

**Fig 2:**
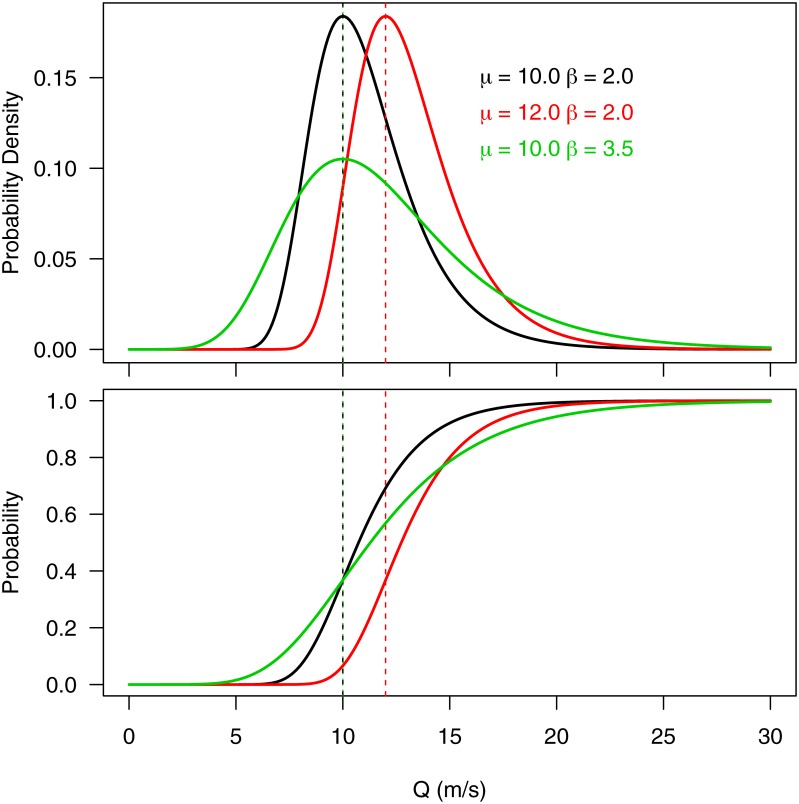
The probability density function (top) and cumulative distribution function (bottom) of the Gumbel distribution [22] (Eqs 2 and 3). The black and red curves show the effect of an increasing *mu*, whereas the black and green curves show the effect of an increasing *β*. An increase in *μ* is associated with an increase in magnitude, while an increase in *β* is linked to an increase in the range.

Climate change induced differences were investigated by comparing the A1B and A2 modeled discharge series with the baseline. Baseline scenarios were generated by GCMs using historical atmospheric conditions. Comparison between the baseline and scenarios was performed by fitting a Gumbel distribution through the modelled runoff (Q) series and then extracting the Gumbel parameters [22]. Fig 2 shows the probability density function (pdf) and cumulative distribution function (cdf) of three hypothetical Gumbel distribution functions. These were calculated using Eq 2 (cdf) and Eq 3 (pdf), where *μ* represents the mode, *β* a scale parameter and the median (*μ*_1/2_) is given by *μ* − *β* *ln*(*ln*(2)). The three curves in Fig 2, show that increases in *μ* results in higher discharge (Q), while an increase in *β* results in a larger range. Thus, a change in *μ*_1/2_ or *β* indicates a change in river flow magnitude or river flow variability, respectively.

$$f\left(Q\right)=\frac{1}{\beta }e^{-\frac{Q\;-\;\mu }{\beta }\;+\;e^{-\frac{Q\;-\;\mu }{\beta }}}$$ (2)

$$F\left(Q\right)=e^{-e^{-\frac{x\;-\;\mu }{\beta }}}$$ (3)

The probability of a specific extreme event can be calculated using the cumulative probability (F(Q): Eq 3) can be expressed by its return period y (Eq 4). As such, the maximum flood (Q) expected within a given number of years (y) can be expressed by Eq 5.

$$y=\frac{1}{1-F(Q)}$$ (4)

$$Q=-ln(-ln\left(1-\frac{1}{y}\right))*\beta +\mu$$ (5)

## Results

**Fig 3:**
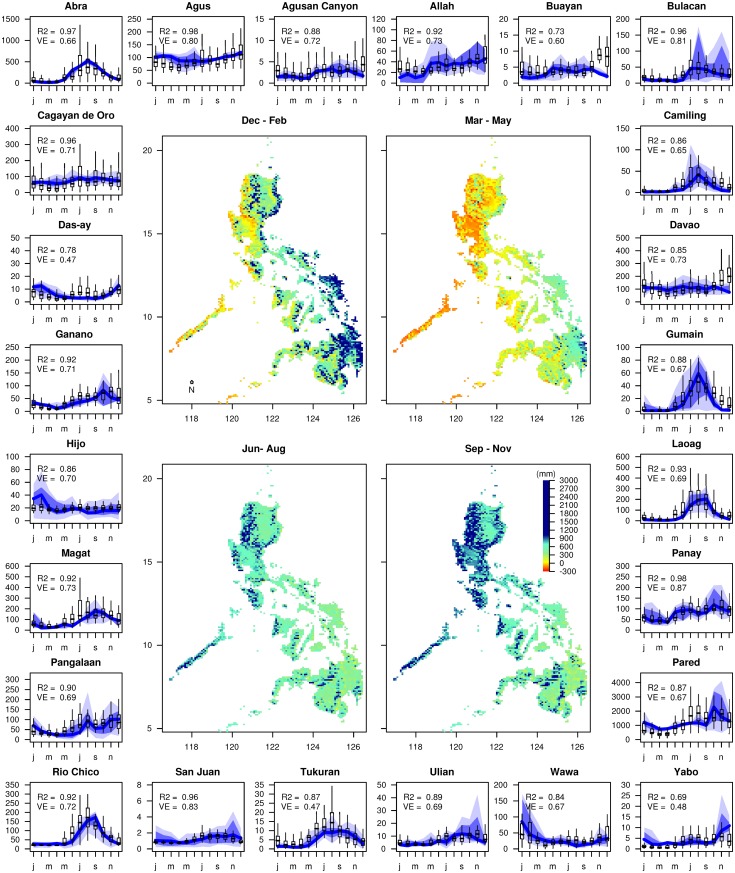
Water balance for the four different seasons according to the interpolated Eraint dataset is shown in the middle. The four figures show the water balance for Dec-Feb (top-left), Mar-May (top-right), Jun-Aug (bottom-left) and Sep-Nov (bottom-right). Surrounding this are the plotted model calibration results that compares the measured data series (blue shading) with modelled ones (box-plots). The blue lines represent the median of the measured data series, whereas the blue shaded areas represent the inter-quartile range. *R*^2^ and volumetric efficiency (*VE*) are also presented in each of the plots.

The results of the STREAM calibrations and seasonal water availability are shown in Fig 3, where the downscaled average (1979-2010) seasonal water balances (precipitation-evaporation) are shown for four different periods (Dec-Feb, Mar-May, Jun-Aug and Sep-Nov) across the Philippines, with values mostly positive, and ranging up to 3000 mm. For climate Types I and III, values are mostly negative during the dry season, which means water from soil storage is mostly evaporated. The different climate types (see Fig 1) are well represented, with relatively dry periods for Types I and III in the months Dec-May (two left figures in Fig 3), whereas Type II is relatively wet during these periods. For climate Type IV, the water balance is relatively even throughout the year, while water availability is higher in mountainous regions compared to lower elevation areas.

Calibration results of the hydrological simulations for the 24 basins are shown on the border of Fig 3. The blue line represents the monthly median of measured runoff values, the shaded areas are the inter-quartile ranges, while the box-plots show the distribution of modelled runoff. For all basins, there is close agreement between simulated and measured runoff values in terms of magnitude, but also seasonal streamflow patterns which are well represented. Correlations between the monthly median measured and simulated runoff, range between *R*^2^ = 0.60 for Gumain to *R*^2^ = 0.98 for Agus and Panay. There is a large variation in *VE* (Eq 1), which ranges from 0.47 for Tukuran, to a maximum of 0.87 for Panay. Distribution deviations often result from limited measured river flow data, as these small datasets do not include the wider distribution of river flows. Given the strength of the predicted vs. observed data for water quantity and seasonality in Fig 3, there is sufficient confidence for using the same set of parameters to simulate the different GCM scenarios.

**Fig 4:**
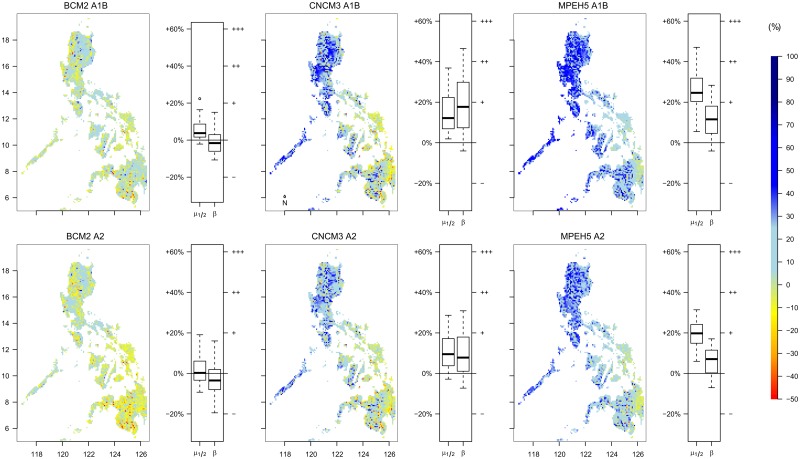
Yearly average water balance produced by the BCM2, CNCM3 and MPEH5 GCMs for A1B (top row) and A2 (bottom row) scenarios in comparison with the baseline. A positive number (%) indicates an increase of available water, a negative number indicates a decrease. The (*μ*_1/2_) and *β* of the Gumbel distribution were determined for all basins using Eq 3. An increase or decrease in median streamflow compared to the baseline is displayed with a percentage in the box-plot, while an increase (+) or decrease (-) in variability (*β*) is also shown in the box-plot.

Fig 4 compares the yearly averaged water balance (PREC-PET) of the six scenarios (maps) with the baseline (as a percentage), which reveals an increase in water for most parts of the country for both scenarios. However, the A1B displays a larger increase compared to the A2 scenario. The GCMs also highlight a difference in water quantity. The largest increase was found using MPEH5, followed by CNCM3 and BCM2 (Fig 4). Spatial differences are also evident and are consistent for all GCMs and all scenarios. In the northern part of the country, the highest increase in water availability was expected. When moving south, water excess decreases, including an expected decrease in water for parts of Mindanao. Patterns of increase or decrease do not seem related to specific climate types, except for climate Type II, which shows consistently lower values compared to the rest of the country (refer to Figs 1 and 4).

To evaluate changes in the amount and variability of river flow, the calibrated model was used to determine *μ*_1/2_ and *β* (Eq 2), which represents stream flow magnitude and stream flow variability, respectively. For all basins, we found a relationship of *R*^2^ > 0.98 when using non-linear regression. Next, the values of *μ*_1/2_ and *β* were compared with the baseline scenario. The box-plots of Fig 4 show the results for each scenario. The BCM2 model predicts stream flow volumes and variability comparable with the current situation, but with a decrease in water availability for some basins. The CNCM3 and MPEH5 models reveal an increase in river discharge volumes and variability, which are both larger for the A1B scenario compared to A2. At the yearly timescale, an increase in both volume and variability would be expected for most basins.

**Fig 5:**
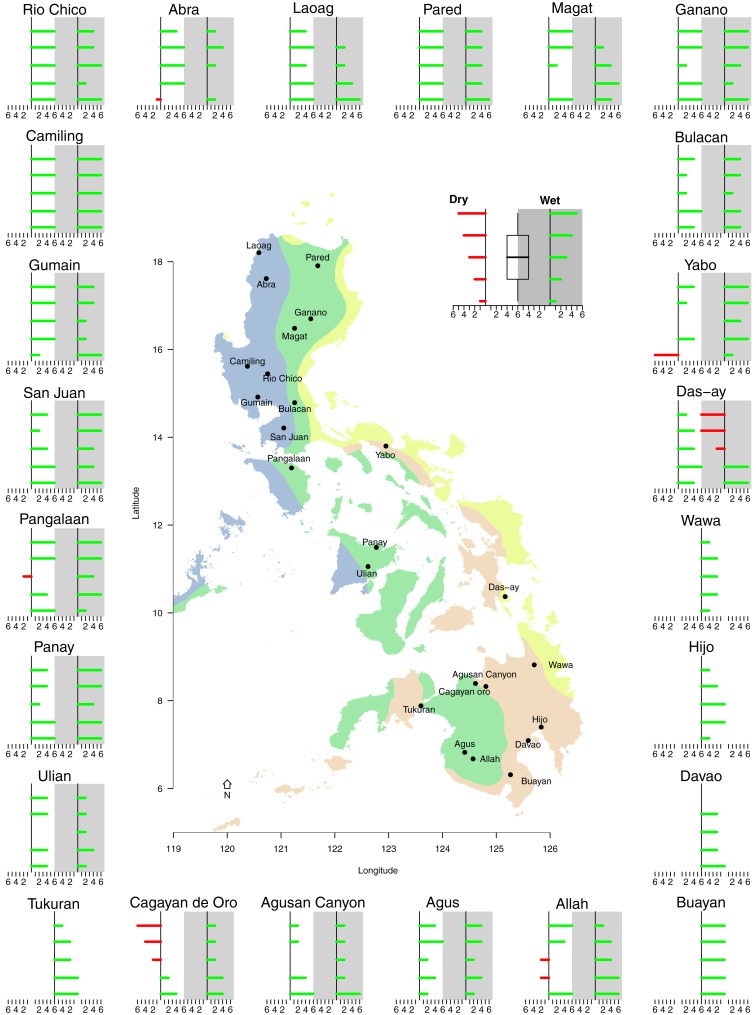
For each basin under each scenario, the minimum, median, maximum and upper and lower quintiles were calculated and compared with the baseline scenario. An increase in streamflow was indicated with a green bar and a decrease with a red bar. The different lengths of the horizontal bars indicate the number of scenarios with an increase or decrease for the dry (left) and wet (right) season.

Given that a redistribution of water between the dry and wet season might adversely affect agricultural practices, the seasonal variability of river runoff was studied in more detail. For the six different GCM-scenario combinations, the minimum, median, maximum and inter-quartiles of river discharge were calculated for the dry and wet season. Fig 5 reveals if these values increased (green bar) or decreased (red bar) compared to the baseline scenario for the dry (left) and wet (right) season. The size of the bar indicates the number of scenarios that experience an increase or decrease, respectively, whereas no bar indicates an equal number of scenarios that increased and decreased. For climate Type IV, the year round results were compared, as this zone has no distinct wet season. The wet season includes maximum rain periods for Type I (May-Oct) and Type II (Nov-Jan). For climate Type III we used the relatively dry period from Nov to Apr to distinguish between wet and dry. A very consistent overall increase in streamflow is predicted by all GCMs and all scenarios for the whole country in both the dry and wet season. The scenarios show that the most consistent increase was for Luzon in the wet and dry season, whereas results for Visayas and Mindanao in the dry season are less consistent. No clear trend was found when comparing the different climatic types. In general, there was an overall increase in water availability for the wet and dry season in the Philippines. The smallest increase, and potentially a projected decrease in current rainfall, was observed in the easternmost regions of Visayas and Mindanao for all model runs.

**Fig 6:**
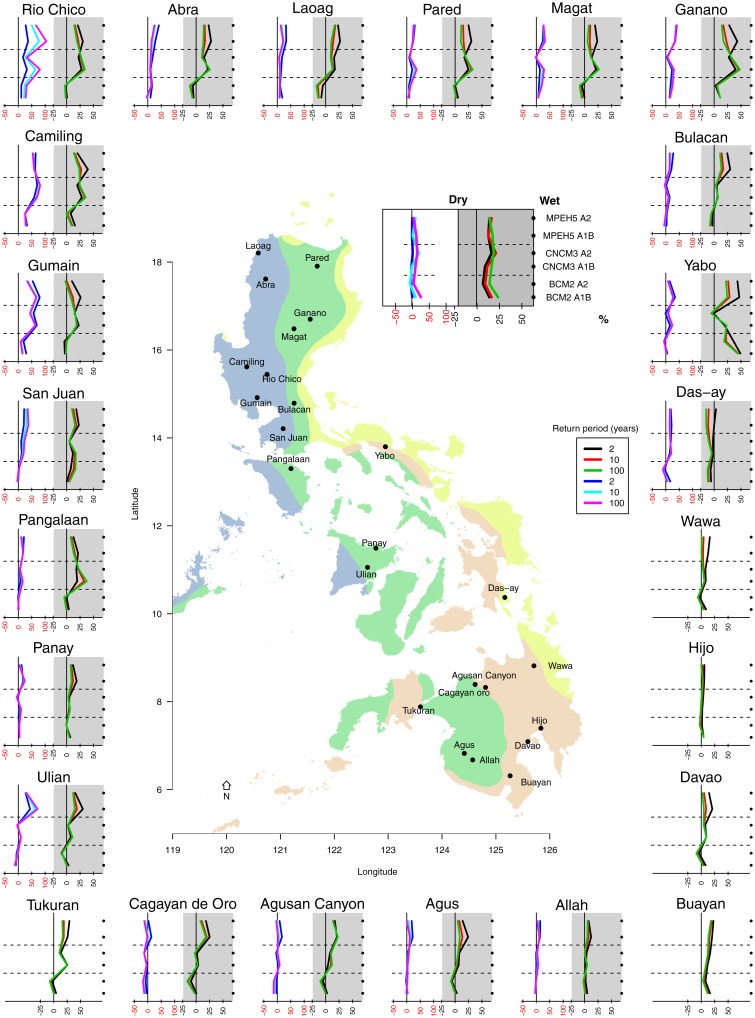
The 2 (black, blue), 10 (red, turquoise) and 100 (green, pink) year return flows were calculated for all scenarios in the 24 basins. An increase or decrease in return flow is expressed as a percentage compared to the baseline scenario. Return flows for the dry period are shown in the white box and return periods of the wet season in the gray box (note the difference in scale). The dots on the right of each plot indicate the GCM and scenario used (refer to the legend).

A general increase in water availability might be beneficial for the agricultural sector but there are other potential problems in the form of flooding during intense rainfall and higher peak flows. Therefore, the return periods (2, 10, 100 years; Eq 5) of peak flows were investigated and compared with baseline scenarios. Fig 6 reveals the percentage increase or decrease of return flows for each basin in the dry and wet season. For the dry season, an increase in peak flows were found for the CNCM3 and MPEH5 models in Luzon, whereas the magnitude of return flows would remain unchanged according to the BCM2 model. It was also expected that Luzon will experience the most dramatic increase in peak flows for all scenarios. Luzon and Viscayas can expect an increase in return flow compared to the baseline in the dry and wet season (see Fig 6). For Visayas and Mindanao, return periods are predicted to increase in the dry season, but remain similar to the current wet season.

## Discussion

The water balance calculations show an overall increase in water quantity for both the wet and dry seasons with river flow distributions revealing an overall increase in water availability. The data reveals a clear north-south trend rather than a trend associated with different climatic types. The northern part of the country (Luzon) is expected to experience a dramatic increase in peak river runoff, while Visayas and Mindanao are predicted to experience an increase in river discharge that is not associated with higher peak flows. Areas with climate Type IV will experience the least impact from climate change, apart from a small increase in water quantity and return periods of high water levels. However, due to data scarcity, hydrological simulations were only performed for three basins in Visayas and nine in Mindanao. Moreover, no representative basin from climate Type II was included.

The projected increase in water availability would benefit the agricultural sector, especially in the dry season when water is scarce. Furthermore, these near-future projections show a reduction in vulnerability to drought of rainfed cropping systems, which may facilitate the expansion of the current irrigated cropping systems in the wet season. However, the large increase in return floods in Luzon might jeopardize this advantage. An increase in extreme rainfall events will aggravate water induced soil erosion in highland areas [23], especially at the onset of the growing season when fields are bare. Low-lying areas on the other hand will be exposed to increased flood risk. The order of magnitude of this flood risk is especially concerning. Given these projections, it is evident that adaptation measures for flood control such as sustainable land management, ecosystem services and infrastructural works are necessary to reduce climate change impacts.

This study provides a general assessment of plausible climate change impacts on hydrology and water resources in the Philippines. However, for the available data, the relatively coarse scale of the climate data and the nationwide approach, specific aspects such as land cover changes or hydraulic infrastructure that might significantly impact local hydrology were not explicitly accounted for in this study. The hydrology model is a simple water balance model without sophisticated water routing. The estimations presented of river flows are at the basin outlet but does not include spatio-temporal dynamics within the basin. Important climatological features such as tropical cyclones and El Ni $\tilde{n}$ o and La Ni $\tilde{n}$ a events were also not specifically considered in this study. The effects associated with these events are embedded in the GCMs and cannot be accounted for seperately. A more direct link between the climate models, downscaling techniques and hydrological model would facilitate the investigation of a wider range of options.

Future studies might utilize a similar methodology to study the impacts of climate change on water resources for a wider array of climate scenarios and downscaling techniques using the next generation of climate models. Results for specific basins should be interpreted as a trend rather than absolute changes, as GCMs have some inherent uncertainties. While the downscaled climate projections include many of the local climatological features, they can still be considered as rather coarse from a hydrological perspective. However, it is reassuring that closely located basins show similar trends in terms of changes in magnitude and variability.

## Conclusion

Three GCMs with two different scenarios each show a clear increase in river flows for the wet and dry season in the Philippines. While the minimum river flow levels were found to increase, so did flow variability, due largely to higher magnitude maximum flows. Climate change effects for Visayas and Mindanao are expected to be relatively mild compared to Luzon, where a dramatic increase in return intervals for maximum river flow rates is predicted. Benefits of an increase in availability of water resources could be jeopardized by pronounced water-induced soil erosion and enhanced risks of future floods.
